# Online questionnaire, clinical and biomechanical measurements for outcome prediction of plantar heel pain: feasibility for a cohort study

**DOI:** 10.1186/s13047-021-00472-w

**Published:** 2021-04-26

**Authors:** Halime Gulle, Trevor Prior, Stuart Miller, Aleksandra V. Birn-Jeffery, Dylan Morrissey

**Affiliations:** 1 Sports and Exercise Medicine, William Harvey Research Institute, Bart’s and the London School of Medicine and Dentistry, Queen Mary University of London, Mile End Hospital, Bancroft road, London, E1 4DG UK; 2 Consultant Podiatric Surgeon, Homerton University Hospital, Homerton Row, London, E9 6SR UK; 3 School of Engineering and Materials Science, Institute of Bioengineering, Queen Mary University London, Mile End, Bancroft road, London, E1 4DG, UK; 4 Physiotherapy Department, Barts Health NHS Trust, London, UK

**Keywords:** Online questionnaire, Graded loading challenge, Feasibility, Cohort

## Abstract

**Background:**

Plantar heel pain (PHP) accounts for 11–15% of foot symptoms requiring professional care in adults. Recovery is variable, with no robust prognostic guides for sufferers, clinicians or researchers. Therefore, we aimed to determine the validity, reliability and feasibility of questionnaire, clinical and biomechanical measures selected to generate a prognostic model in a subsequent cohort study.

**Methods:**

Thirty-six people (19 females & 17 males; 20–63 years) were recruited with equal numbers in each of three groups: people with PHP (PwPHP), other foot pain (PwOP) and healthy (H) controls. Eighteen people performed a questionnaire battery twice in a randomised order to determine online and face-to-face agreement. The remaining 18 completed the online questionnaire once, plus clinical measurements including strength and range of motion, mid-foot mobility, palpation and ultrasound assessment of plantar fascia. Nine of the same people underwent biomechanical assessment in the form of a graded loaded challenge augmenting walking with added external weight and amended step length on two occasions. Outcome measures were (1) feasibility of the data collection procedure, measurement time and other feedback; (2) establishing equivalence to usual procedures for the questionnaire battery; known-group validity for clinical and imaging measures; and initial validation and reliability of biomechanical measures.

**Results:**

There were no systematic differences between online and face-to-face administration of questionnaires (*p-values all > .05*) nor an administration order effect (d = − 0.31–0.25). Questionnaire reliability was good or excellent (ICC_2,1_absolute_)(ICC 0.86–0.99), except for two subscales. Full completion of the survey took 29 ± 14 min. Clinically, PwPHP had significantly less ankle-dorsiflexion and hip internal-rotation compared to healthy controls [mean (±SD) for PwPHP-PwOP-H = 14°(±6)-18°(±8)-28°(±10); 43°(±4)- 45°(±9)-57°(±12) respectively; *p < .02* for both]. Plantar fascia thickness was significantly higher in PwPHP (3.6(0.4) mm vs 2.9(0.4) mm, *p = .01*) than the other groups. The graded loading challenge demonstrated progressively increasing ground reaction forces.

**Conclusion:**

Online questionnaire administration was valid therefore facilitating large cohort recruitment and being relevant to remote service evaluation and research. The physical and ultrasound examination revealed the expected differences between groups, while the graded loaded challenge progressively increases load and warrants future research. Clinician and researchers can be confident about these methodological approaches and the cohort study, from which useful clinical tools should result, is feasible.

**Level of evidence:**

IV

**Supplementary Information:**

The online version contains supplementary material available at 10.1186/s13047-021-00472-w.

## Introduction

Plantar heel pain (PHP) is one of the most common foot and ankle problems, causing pain on the plantar aspect of the rear-foot, particularly at the inferio-medial heel and accounting for approximately 11–15% of all foot symptoms requiring professional care [1]. People with PHP (PwPHP) often complain that the most severe pain occurs during the initial step, after a period of prolonged non weight-bearing [2]. The course of the disease has long been regarded as self-limiting but this is now known not to be the case [2].

Various treatment strategies are proposed for PwPHP, but outcomes are not satisfactory, with no accepted treatment of choice [3] and no clear prognostic indicators. Recovery rates from the many tested interventions vary between 50 and 80% at 6 months [4]. Footwear modification, foot orthosis, taping, stretching and shockwave therapy (ESWT) have the best evidence for managing PHP [5, 6]. However, approximately 50% of individuals continue to have some symptoms after conservative treatment and at least 30% have recurrent symptoms [7]. The associated factors relevant to prognosis are thought to be a high body mass index (BMI) or sudden weight gain, excessive running, prolonged standing/walking, occupational environment, work-related weight bearing activities, limited ankle dorsiflexion, a cavus foot, excessive foot pronation and psychological symptoms (e.g., depression, anxiety, and stress) [8, 9]. However, the prognostic evidence of these factors is neither complete nor causal [3].

Prospective research for PwPHP has typically considered single or limited numbers of outcome predictors with analysis limited by relatively small sample sizes [3, 10]. Although numerous studies using cross-sectional or matched case-control designs have been conducted [11, 12], at best single variable prediction models have been created [7]. In order to increase treatment success enabling prognosis determination could be helpful by taking multiple factors into consideration as in case for other pathologies. For example, prognostic screening tool such as the StartBack, which is an easily completed multiple scale that combines potentially modifiable prognostic factors including pain, function and fear avoidance behaviour, can increase health benefit and yield cost savings for low back pain [13]. Therefore, high-quality prospective cohort studies with a large sample size are needed to identify the relative importance of multiple outcome predictors. The impact of revealing these outcome predictors would be useful to clinicians judging prognosis, researchers who want to understand causal relationships and perhaps for sufferers seeking to understand their condition if presented in suitable translational materials. Multi-variable models that perform better than single variables or overall clinician judgement of outcome would be of particular use [14], with a planned cohort study having been designed to build an accurate prognostic model for PHP outcome. Importantly, it may be that the model is specific to PHP but not other foot pain (OP), and so the investigation of people with other foot problems is needed to compare the two and determine factors that are specific to PHP.

We judged that an online questionnaire approach would enable easier access to more participants in a wider variety of locations at lower cost. The advantages of online delivery were central to maximising cohort study recruitment, but modifications applied require validation compared to the original paper version of the questionnaires according to ISPOR ePRO guidelines [15]. These stipulate that moderate modifications require validation hence, as we combined numerous PROMs into a questionnaire battery within a complex study design with various formatting changes, it was essential to perform an equivalence study.

Therefore this study primarily aimed to investigate feasibility by testing data collection procedures and gaining feedback from participants in order to refine data collection. Establishing equivalence to usual procedures for the questionnaire battery; known-group validity for clinical and imaging measures; and initial validation and reliability of biomechanical measures in the form of a graded loading challenge were secondary aims. These data were required in order to optimise the success of a prospective cohort study.

## Methods

### Study population

A convenience sample of thirty-six participants with equal numbers of people with PHP, people with other foot pain (PwOP) and healthy controls were recruited from private clinics and local facilities in London, UK from an initial sample of 48 over a three month period in 2018. The inclusion criteria were a diagnosis of PHP for the PHP group and a different diagnosis of an ankle or foot musculoskeletal condition for the PwOP group. A podiatrist with over 30 years’ clinic experience (TP) diagnosed both groups of conditions based on reported symptoms, clinical examination; subjects with early morning and first step pain for more than one month and pain on palpation of the plantar medial tubercle of the calcaneus were classified as people with PHP compared to other foot problems [16]. Healthy controls were defined as not having any foot and ankle related problems before. People under 18 years of age were the only exclusion.

The study procedures were ethically approved by QMERC ethics committee (approval No. QMREC2014/24/153). Written informed consent was sought from each recruited participant prior to study entry either via the online questionnaire or face-to-face. The consort-PF [17] guidelines were consulted to guide study design.

### Measures

#### Questionnaire battery

An online survey was constructed and administered using ‘SurveyMonkey’ (www.surveymonkey.com). The standard patient reported outcome measures (PROMs) format was reproduced as closely as possible using the same wording of the items and instructions. The online survey consisted of eight PROMs and miscellaneous questions designed to collect outcome measures, consisting of pain severity, restriction level of some activities, kinesiophobia, and report of pain location with a pain map, physical activity level, quality of life, age and BMI, which are all considered as relevant factors for prediction of PHP prognosis.

The Foot and Ankle Outcome Score (FAOS) was used to assess foot and ankle problem severity, activity limitation, and participation restriction [16, 18]. The FAOS is an adaptation of the KOOS and consists of 42 questions with five subscales: pain (nine questions); symptoms (seven questions); activities of daily living and limitations (17 questions); ability to perform sports and recreational activities (five questions); and quality of life related foot/ankle (four questions). The score is calculated by summing the scores of the individual items. The total score is on a 0–100 scale, with 100 representing no symptoms or limitations [18]. The validity and reliability of the original FAOS, as well as other different translated versions, is considered good [18, 19].

Psychological variables are common in people with chronic musculoskeletal pain and are associated with pain and function [20] Those psychosocial features were evaluated by the Pain Catastrophizing Scale (PCS) and Fear-Avoidance Belief Questionnaire (FABQ) [21]. PCS was used to measure pain-related catastrophizing with 13 items that yield an overall score [21] which greater than 24 have been associated with higher catastrophization [22]. Reliability and validity of the PCS have been established [21, 23, 24]. FABQ is designed to assess fear of avoidance beliefs on movement for patients with musculoskeletal condition and chronic pain [25]. The questionnaire consists of two subscales that relate to work (7 questions) and physical activity (4 questions) with 7-point Likert scales. Higher values indicate a greater fear of movement. The FABQ demonstrates high levels of internal consistency and test-retest reliability [26–28].

Evidence suggests that a history of occupational/daily activities involving long periods of standing or inactivity may be associated with PHP [16, 29]. Physical activity was assessed with the Global Physical Activity Questionnaire (GPAQ) [30]. The PAQ comprises 16 items that measure physical activity in work, transport, leisure activities, and time spent in inactivity by measuring intensity, duration, and frequency. The GPAQ showed acceptable evidence of short- and long-term test–retest reliability by activity category and modest validity evidence [31].

Additionally, PHP has a significant negative impact on foot-specific and general health-related quality of life, itself assessed by using the Euro quality of life (Euroqol) five dimension 5 level questionnaire (EQ-5D-5L) [32, 33]. EQ-5D-5L measures generic health status by taking into account five dimensions; mobility, self-care, usual activities, pain/discomfort, and anxiety/depression. Total score can be converted into a single preference-based index anchored on a scale where 0 and 1 represent being dead and full health, respectively [34].

#### Clinical examination & ultrasound assessment

A subset of eighteen participants underwent a lower-extremity physical examination by a physiotherapist, consisting of selected clinical measures based on clinical practice guideline [2, 16] and clinical experience indicating relevance to prognosis. These measures included lower limb strength of gastrocnemius and hip extensors and hip internal rotation and ankle dorsiflexion and MTPJ1 dorsi flexion range of motion measures [16, 35–37]. Mid-foot mobility was measured via navicular drift, navicular drop and medial longitudinal arc (MLA) angle [38, 39]. Finally, we palpated the midpoint of the heel, medial insertion of plantar fascia and insertion of Achilles tendon and gastrocnemius muscle belly to detect painful areas [2].

Ultrasound scanning (US) was used to examine the plantar fascia at its origin and mid-section, with long-axis sonograms using a 7.5 MHz probe (GE Logiq S8, Milwaukee, WI, USA). Heel pad thickness, echogenicity, bony erosions, heel spurs, ossification, and signs of fascia rupture or fibroma were sought as reduced fascia thickness and other US findings could also be a sign of PHP recovery [3]. Neovascularization was graded using a modified Ohberg grading scale from 0 to 5 [40].

#### Biomechanical assessment

Biomechanical assessment was performed twice (2–7 days between tests) with a subset of nine participants. A graded loading challenge (GLC) was developed to assess pain response and movement features in response to increasing step length and weight carried. The test consisted of four different difficulty levels: 1) normal walking with self-selected speed and step length, 2) walking with a 25% longer step length of participants’ original step, 3) normal walking while carrying a load of 25% of body mass (BM), and 4) walking with the 25% longer step length plus the extra 25% load, which is a combination of tasks two and three. Participants performed each level 10 times, with each repetition consisting of six (level 1 and 3) or four (level 2 and 4) steps prior to the force plate and the same number of steps after; the total walking distance of walking was approximately 11 m. Participants carried load via a double-sided weighted vest (HOMCOM, MHSTAR, England). Step length was guided by indicators of the individually-determined required step length on the ground.

Kinetic and kinematic motion capture were performed during the GLC utilising in-floor force plates (500 Hz; 9281CA, Kistler) and an infrared motion analysis system (100 Hz; CX-1, Codamotion, Charnwood Dynamics Limited, Leicestershire, UK), respectively. Thirty-four infrared markers were used, consisting of 14 individual markers on foot anatomical landmarks using Leardini protocol [41], four rigid clusters of four markers placed bilaterally on shank and thigh, and four markers located on the anterior and posterior superior iliac spine.

#### Validity, reliability and feasibility of procedures

**Fig. 1:**
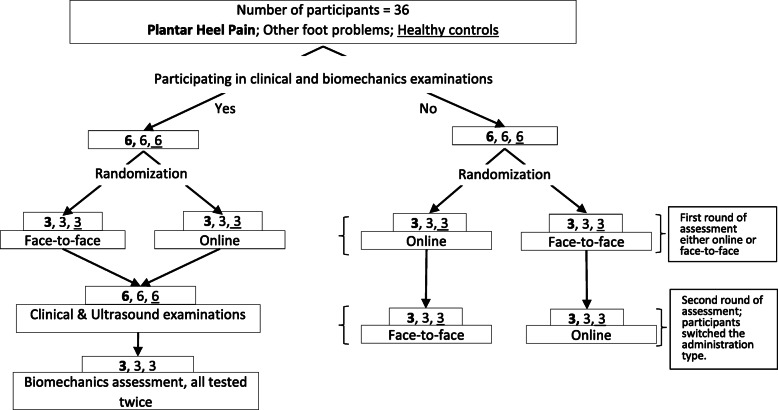
Feasibility study design with randomization

Thirty-six participants were divided into two groups based on willingness to participate in the clinical and biomechanical examinations (Fig. 1, left arm). Group one (eighteen participants) undertook the questionnaire clinical and ultrasound measures – with a subset of nine performing the biomechanical measures on two occasions (second aim). Group two (the remaining 18 participants) undertook the questionnaire battery both online and face-to-face in a randomised order (Fig. 1, right arm) to assess validity and reliability of online questionnaire (first aim).

### Validity

#### Questionnaire validity

To assess the validity of delivering the questionnaires online, the delivery was conducted online and face-to-face in a randomised order. Randomisation was conducted by an independent person not otherwise involved in the study, using an online true random-number service (www.random.org).

#### Clinical and biomechanical validity

Validity of the clinical and biomechanical measurements was assessed utilising known-group validity (I.e. ability to detect differences between the three groups). This approach was considered to allow selection of useful measures for the proposed cohort study.

### Reliability

Survey reliability was evaluated by testing the consistency of measures regardless of administration type. Biomechanical measures were compared between the two testing sessions for consistency. Re-tests were implemented between 2 and 7 days.

### Feasibility

Feasibility was assessed by completion time and feedback from participants/assessor.

### Calculation of sample size

The sample size was calculated separately for validity and reliability. Validity sample size was calculated using G*Power (version 3.1), based on the FAOS foot function subscale. According to previous studies showing mean scores of 57.8 ± 24.4, 74.61 ± 21.94, 96.1 ± 12.4 for PwPHP, PwOP and C, respectively [33, 42], a minimum of 18 participants was required for validity based on 90% power, and an α level of 0.05. Sample size calculation for reliability was based on ICC values. A method that explicitly incorporates a prespecified probability of achieving the prespecified width or lower limit of a confidence interval was utilized [43]. This resulted in 14 participants being required based on ICC limits of 0.6 and 0.9. A final sample size of 36 participants was determined, consisting of 18 for validity, reliability and for feasibility [35].

### Data analysis

A list of all the measures (battery of questionnaires, and clinical and biomechanical assessments) is shown in Table 1 (results section).

**Table 1:** Sample Characteristics

Demographics	Plantar Heel Pain (*n* = 12)	Other Foot Problems (*n* = 12)	Healthy Controls (*n* = 12)
Gender (female:male)	6:6	6:6	7:5
Age, years (mean ± SD)	41 ± 16*	38 ± 13	28 ± 2.7
BMI, kg/m^2^ (mean ± SD)	27 ± 5.2^$^*	24 ± 3.9	23 ± 3.1
Morning Pain Severity, VAS (mean ± SD)	43 ± 20	54 ± 14	NA
Morning Pain Duration, mins. (mean ± SD)	24 ± 18	25 ± 19	NA
FAOS (mean ± SD)	55 ± 28^$*^	80 ± 17	99 ± 1
Occupation, (n, (%))			
Blue-collar	0 (0%)	0 (0%)	2 (17%)
White collar	10 (83%)	9 (75%)	6 (50%)
Unemployment & students	2 (17%)	3 (25%)	4 (33%)
Exercising regularly, (yes:no)	(9:3)	(9:3)	(7:5)

*P*-values for differences in means between groups calculated using Kruskal Wallis. *p < 0.05 compared to healthy controls, $*p* < 0.05 compared to other foot problems. Key = *n* number of participants, *kg* kilogram, *m* metre, *BMI* Body Mass Index, *mins* minutes, *VAS* Visual analogue scale, *FAOS* Foot and Ankle Outcome Score)

To allow for ease of comparison and presentation of findings across different PROMs, all scores were adjusted to a scale of 0–100 if necessary. Specifically, the GPAQ, FABQ and PCS scores were multiplied by a hundred, and then divided by the maximum score possible on the scale.

To assess reliability of the pain maps, participant-selected locations were marked with 1 if they matched, and 0 if they did not, with unselected locations also counted as matching; total percentage similarity was then used for reliability.

Biomechanical data was processed and analysed using custom-written scripts in MATLAB version R2018b (Mathworks, Natick, MA). Force plate data were low-pass filtered (Butterworth, 6th-order and cut-off frequency of 10 Hz). The peak vertical ground reaction force (vGRF) at loading response (first peak) and terminal stance (second peak) were selected based on previous research [44]. Kinematic marker data were low-pass filtered (Butterworth, 4th-order and cut-off frequency of 12 Hz). Medial longitudinal arch (MLA) and first metatarsophalangeal joint (MTPJ1) angles were analysed at 50% stance and toe off, respectively. Toe off was identified using the markers on the MTPJ1, hallux and navicular bones, verified with vertical GRF. Both kinematic variables were calculated in sagittal plane [41].

### Statistical analysis

For validity of online delivery, differences between online and face-to-face questionnaires were tested using Limits of Agreement with Bland & Altman plots [45] and paired t-test, considering order effect. Cohen d statistic was used to show the magnitudes of differences between two modes. Cohen’s d was interpreted as, 0.20 < d < = 0.50 indicated a “small effect”, 0.50 < d < = 0.80 a “medium effect”, and d > 0.80 a “large effect” [46]. Mann-Whitney U test with Bonferroni correction were used to assess differences between groups for clinical and US examinations. Graded Loading Challenge values were analysed with Repeated Measures. Reliability was determined with Intraclass Correlation Coefficients (ICC, two-way random, absolute agreement), classified as < 0.5, 0.5 to 0.75, 0.75 to 0.9, and > 0.90 being poor, moderate, good, and excellent reliability, respectively [47]. Outliers were removed if they were not within three standard deviations (μ ± 3σ) [48]. All data were analysed using Microsoft Excel Version 2013 (Microsoft, California, USA) and SPSS Version 24.0 (SPSS, Chicago, IL).

## Results

### Sample characterisation

Recruitment continued until there were the required numbers for the study arms (Fig. 1). Forty-eight participants were eligible and consented to join the study, half beginning with the face-to-face questionnaire and half online. All face-to-face questionnaires were completed. Three did not complete the initial online questionnaire and 9 did not complete it in the second round giving 66 complete questionnaire battery responses out of 78, a completion rate of 94% (45 of 48) in round 1 and 80% of online questionnaires in round 2 (36 of 45). The data for the 36 people (19 females & 17 males) who completed both rounds were analysed with equal numbers in each of the three groups: people with PHP (PwPHP), other foot pain (PwOP) and healthy (H) controls. Participants both groups had similar sample characteristics (Table 1 and Table 2).

**Table 2:** Values for all measures are reported with validity, reliability and feasibility outcomes

MEASUREMENTS	DOMAIN	PURPOSE	RESULTS	OUTCOMES
***Patient Reported Outcome Measures (n = 36)***				
Pain Catastrophizing Scale (PCS)	Psychosocial factors	V R F	LoA = 0.2 ± 8.5; d = 0.01; *p* = 0.83 Excellent (ICC = 0.97) Patients reported psychosocial questions duplication	Online use valid Reliable measure Redesign order
Global Physical Activity Questionnaire (GPAQ)	Activity level	V R F	LoA = −5.3 ± 22.2 d = − 0.22; *p* = 0.51 Good (ICC = 0.81) Designed logic between relevant question to avoid time wasting and make GPAQ appropriate for online use	Online use valid Reliable measure Time burden Reduction needed
Fear-Avoidance Belief Questionnaire subscale (FABQ)	Psychosocial factors	V R V R F	*PA:* LoA = 1.6 ± 15.9; d = −0.06; p = 0.55 *PA* Excellent (ICC = 0.92) *W:* LoA = − 0.5 ± 8.5; d = 0.25; *p* = 0.77 *W:* Poor (ICC = 0.39) Patients reported psychosocial questions duplication	Online use valid Reliable measure Online use valid Poor reliability Redesign order
Health-related Quality of Life (EQ. 5D-5L)	Quality of Life	V R V R F	*VAS:* LoA = − 0.3 ± 13.6; d = − 0.26; *p* = 0.07 *VAS:* Excellent (ICC = 0.94) *State:* LoA = − 1.1 ± 8.5; 0.16; *p* = 0.55 *State:* Moderate (ICC = 0.64) Easy to report & understandable	Online use valid Reliable measure Online use valid Moderate reliability Easy to use
Foot and Ankle Outcome Score (FAOS)	Physical factors	V R F F	LoA = 1.3 ± 10–2.5 ± 18.2; d = 0.11–0.16 *p* = 0.49–.08 Excellent to moderate (ICC = 0.99–0.73) Patient answers inconsistent for last subscale. Patients reported many questions in physical factors	Online use valid Reliable measure Redesign look Reduce repetition
Key miscellaneous questions	Morning pain duration (mins) Morning pain severity (VAS)	V R V R F	LoA = 2.2 ± 18.7; d = 0.10; p: 0.34 Excellent (ICC = 0.94) LoA = − 2.1 ± 19.0; d = − 0.10; p: 0.33 Excellent (ICC = 0.94) Both measures easy to report & understandable	Overall: Online use valid, reliable measures that are feasible.
Pain map	Foot pain map	V R F	Pain-spreading region with 66% agreement. %98 matched; the medial aspect of RF clumsy system	Valid Use Reliable measure Navigate Pain
***Clinic Examination (N = 18)***				
Foot mobility	Navicular drift Navicular drops MLA angle	V F V F V F	PHP = 6 ± 3; OP = 8 ± 1; H = 7 ± 3 mm; difficult to control medial movement PHP = 10 ± 4; OP = 9 ± 4; H = 12 ± 9 mm; Difficult to determine the change PHP = 160° ± 7; OP = 156° ± 11; H = 155° ± 5 difficult to position and maintain set-up	Overall: a new measurement procedure is required.
Range of motion	Hip IR Ankle active DF 1MTPJ DF	V F V F V F	PHP = †43° ± 4; OP = 45° ± 9; H = 57° ± 12 Difficult to estimate centre of rotation PHP = 27° ± 6; OP = 25° ± 3; H = 27° ± 3 Difficult to estimate true vertical and horizontal positions PHP = 36° ± 4; OP = 38° ± 10; H = 37° ± 7 The test was affected by instrumentation,	Overall: valid measure but binary outcomes needed and amended procedure.
Strength (oxford scale)	H. ER Ankle PF Inversion Intrinsic muscle	V F V F V F V F	PHP = 4.7 ± 4; OP = 4.8 ± 4; H = 5 Difficulty to detect difference between grades PHP = 4.9 ± 2; OP = 4.9 ± 2; H = 5 assesses muscles when contracting concentrically PHP = †3.5 ± 5; OP = 5; H = 5 No difficulty is detected PHP = 4,8 ± 4; OP = 5; H = 4.8 ± 6 Difficulty to control participation of other muscle groups	Overall: valid measure but binary outcome needed and more practical test.
Modified knee to wall	ADROM before NP DFROM in full	V V F	PHP = 20° ± 8; OP = 21° ± 9; H = 21° ± 7 PHP = †14° ± 6; OP = 18° ± 8; H = 28° ± 10 Navicular drop not clear	Overall: sensible values but test needs modified
***Ultrasound Assessment (N = 18)***				
Thickness measures	PF origin Mid PF Heel pad	V V V F	PHP = †^‡^3.7 ± 0.4; OP = 2.6 ± 0.8; H = 2.9 ± 0.4 mm. PHP = †^‡^3.7 ± 0.4; OP = 2.6 ± 0.7; H = 2.8 ± 0.4 mm. PHP = 8.4 ± 0.2; OP = 7.8 ± 0.2; H = 9.3 ± 1.9 mm. Difficult to control pressure	Overall: sensible values but practice needed.
***Biomechanical Assessment (N = 9)***				
Graded loading challenge (GLC)	First vGRF peak (N/BW)	V R F	NW = 7626 ± 1565; LS = 8866 ± 1822; NWW = 9445 ± 1564; LSW = 10,825 ± 1320 Excellent (ICC = 0.92–0.95) Easy to measure & high-quality data	Overall: valid and reliable measure which is feasible to collect.
	Second vGRF Peak (N/BW)	V R F	NW = 7826 ± 1656; LS = 8598 ± 1859; WW = 9569 ± 1541; LSW = 10,919 ± 1805 Good to excellent (ICC = 0.81–0.92) Easy to measure & high-quality data	Overall: valid and reliable measure which is feasible to collect.
	Rate of force development (N. s^− 1^)	V R F	NW = 4741 ± 1307; LS = 5949 ± 1671; WW =5235 ± 1518; LSW =7356 ± 1799 Excellent (ICC = 0.91–0.96) Easy to measure & high-quality data	Overall: valid and reliable measure which is feasible to collect.
	1.MTPJ DF on Toe off phase of gait cycle	V R F	NW = 14° ± 6; LS = 15° ± 7; WW =15° ± 8; LSW =14° ± 6 Moderate (ICC = 0.60–0.71) Time consuming	Sensible values Moderate reliability Discard measure.
	MLA during midstance	V R F	NW = 139° ± 15; LS = 139° ± 15; WW = 140° ± 13; LSW = 143° ± 14 Poor to Good (ICC = 0.53–0.78) Time consuming	Sensible values. Modest reliability Discard measure.

All measurements, their contents, purpose, relative results and outcomes are presented. Results of the clinical, biomechanical and miscellaneous questions are given in three groups to demonstrate differences as mean ± SD. Key: *V* Validity, *R* Reliability, *F* Feasibility, *SD* Standard deviation of mean values, *n* Number of participants, *LoA* Limits of Agreement (mean bias ±1.96*SD); *ICC* Intra-Class Correlation Coefficients, *d* Cohen’s d, *BMI* Body Mass Index, *N* Newton, *BW* Body Weight, *min* minutes, *VAS* visual analogue scale, *ROM* Range of motion, *H.ER* Hip external rotation ROM, *DFROM* Dorsiflexion Range of Motion, *A* Ankle, *ND* Navicular Drop, *1MTPJ* First metatarsophalangeal joint, *PF* Plantar Fascia, *MLA* Medial Longitudinal arch angle, *NW* Normal Walking, *LS* Long-Step walking, *WW* Walking with Weight, *LSW* Long-Step walking with Weight

*†p < .05 compared to control:* ^*‡*^ *p < .05 compared to other foot pain*

### Validity

#### Online survey

**Fig. 2:**
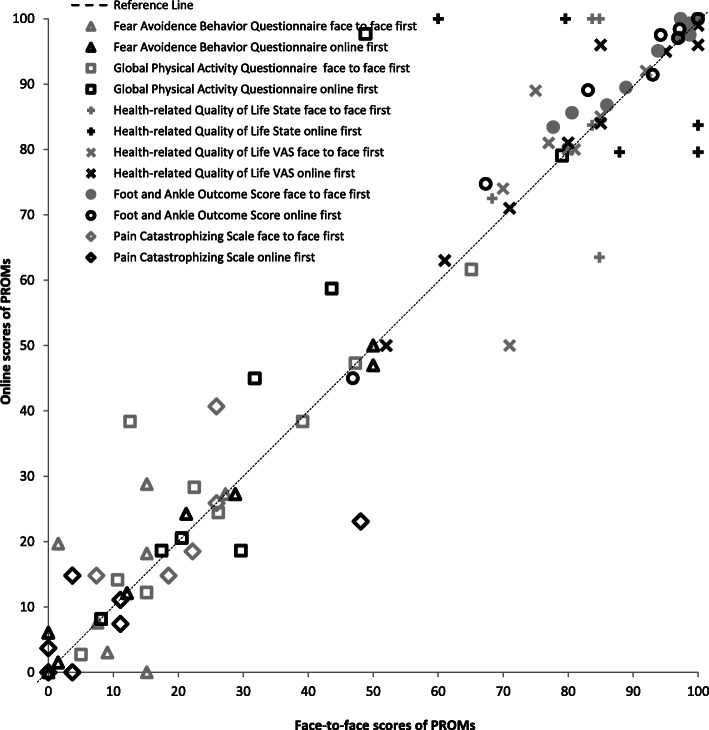
Systematic differences between face-to-face and online administrations

**Fig. 3:**
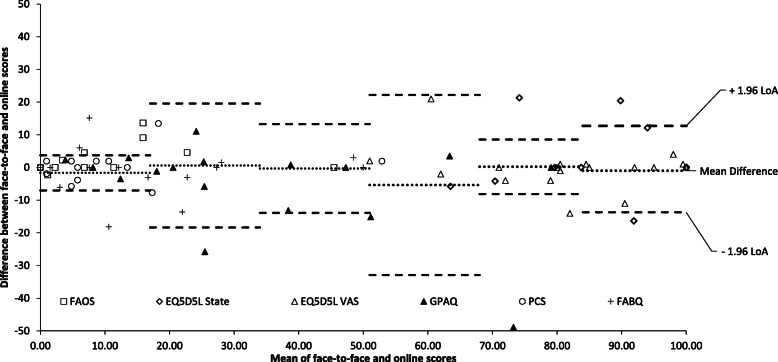
Bland–Altman plot of the relation between face-to-face and online scores of 5 PROMs and 2 subscales

Mean values for all PROMs between online and face-to-face did not differ significantly, (all *p*-values ranged from 0.07 to 0.79; Table 2, Fig. 2, Fig. 3). There were no systematic differences between face-to-face and online methods in terms of administration modes and order (Fig. 3 and Table 2).

#### Clinical examination & ultrasound assessment validity

Clinical assessment showed PwPHP have less active ankle dorsiflexion ROM and hip internal rotation compared to healthy controls (Table 2). In terms of ultrasound findings, both plantar fascia thickness insertion from calcaneus (*p*-value: 0.02) and 0.5 cm away from calcaneal insertion (p-value: 0.03) were significantly higher in PwPHP compare to others.

#### Biomechanical validity

**Fig. 4:**
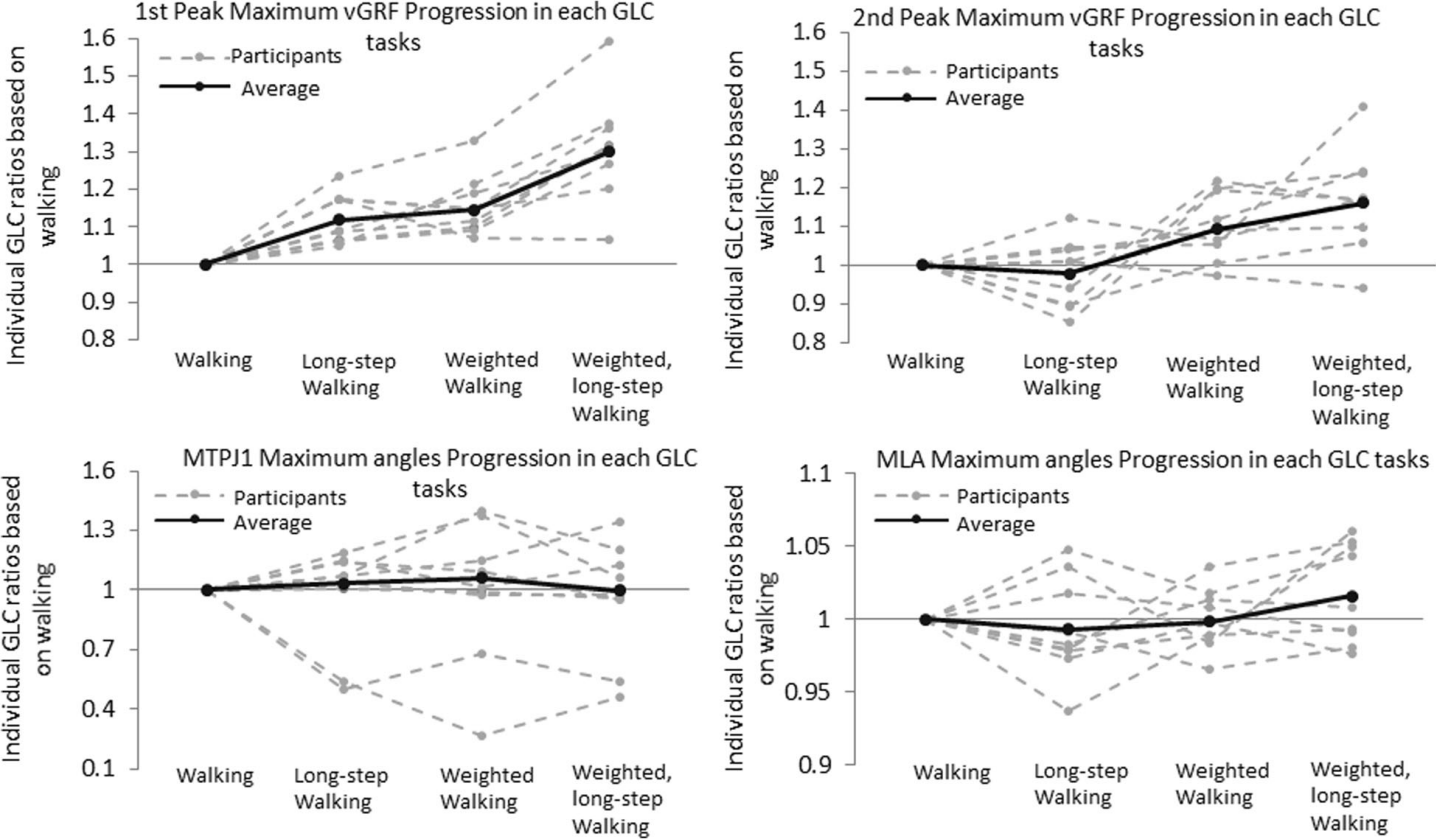
Individual ratio values of 9 participants for biomechanics measures progression in order of GLC tasks

Biomechanical assessment demonstrated the GLC shows increases in maximum (p-value < 0.01) and second peak (p-value < 0.01) of GRFs with no progressive change in kinematics. (Fig. 4 & Table 2).

### Reliability

#### Online survey

Questionnaire reliability was good to excellent (ICC 0.86–0.99) except for two subscales. The quality of life subscale (QoL) of Foot & Ankle Survey (FAOS) had an ICC of 0.73 [^−^ 0.21–0.91] and Fear Avoidance Behaviour Questionnaire (FABQ) work subscale had an ICC of 0.39 [^−^ 0.03–0.77] (Table 2 and Fig. 3). Pain maps were 98% matched between first and second assessments, with eight PwPHP clearly indicating the usual inferior-medial area as painful. Pain map analysis showed the central dorsal rear-foot was the most common painful area with 25% among of all points on the plantar aspect of the foot. Additionally, 66% of participants with PHP identified the medial dorsal rear and mid-foot as a region to which pain spread.

#### Biomechanical reliability

Biomechanical assessment reliability was typically moderate to excellent (ICC 0.60–0.92) except for the MLA within the walking-with-weight task (Table 2).

### Feasibility

#### Online survey

Completion rate was 73% and completion time was 26 ± 14 min. Participants reported the survey to be too long and have some repetition, particularly questions about psychosocial factors. It has been recognized that some terminological words such as “Plantar Heel Pain” need to be well-defined for participant understanding. Moreover, some participants had technical difficulties with the online survey system and were reluctant to share some personal details such as date of birth. Participant feedback details are presented in the supplement.

#### Clinical examination & US assessment

Clinical assessment took an average of 1 h and 25 min. The measures have been streamlined by further practice to improve efficiency.

#### Biomechanics

The kinetic and kinematic motion capture system was found to be a feasible method for measuring the foot and ankle during walking. No subjects reported any discomfort or undesirable effects associated with use of the sensors.

## Discussion

This was a comprehensive validity, reliability and feasibility study designed in order to optimise a large planned prospective cohort study. Importantly, some of the questionnaires had not previously been tested for remote use, but we found the online approach was valid and suitable. A novel graded loading challenge test progressively increased kinetic load and may represent a potentially useful assessment tool for plantar heel pain severity. The validity of clinical, ultrasound and biomechanical measures was confirmed. Reliability of measures was also typically good or excellent. Overall, the measures included in this feasibility study, and the protocols developed, are feasible for the planned cohort study. Key lessons included improving explanation of technical words but otherwise feasibility was acceptable.

### İnterpretation of outcomes

#### Validity

Patient-reported outcome measures (PROMs) are becoming more commonly applied [49] for research health care evaluation purposes, with technology enabling easier access to more participants at lower cost. These advantages are central to maximising cohort study recruitment, but different administration modes require validation compared to the original [50]. In a recent meta-analysis concerning PROMs equivalence between computer and paper versions, the average correlation of 278 PROMs was excellent [51] similar to responses to a comparison across 16 health-related measures [52]. None of the current foot and ankle or more generic PROMS had been previously evaluated [51], but the demonstrated limits of agreement [53] identified no systematic bias and compared well to previously reported questionnaire properties [54]. For example, our FAOS results (LoA = 9.13) compared favourably with published minimally important subscale differences ranging from 5.8 to 11.1 [55], giving confidence about online use. The consistent agreement between methods means that researchers and clinicians can be confident using these methods with similar populations although they may need to consider the particular population of interest and their e-Health literacy level in study or evaluation design [56].

Clinical validity was important to consider, despite established procedures being used that have face validity [16, 57–59]. We assessed whether between-group differences were of similar direction and magnitude to published work, accepting that we had powered the study primarily to assess questionnaire measure validity and the clinical aspects were relatively underpowered meaning differences, or their absence, would have to be interpreted with caution. As expected, PwPHP have less ankle dorsiflexion ROM and hip internal rotation compared to healthy controls (Table 1) which compares favourably with published data [60]. However, our measured differences in first metatarsophalangeal joint movement (36 ± 4° versus 37 ± 7°) were of the same direction but smaller than reported values (46.2 ± 7.3° versus 68.5 ± 13.0°) [60] between PwPHP and control group. Similar to Wearing et al., our plantar fascia thickness measures agreed well. Control group insertion and 0.5 cm distal to the calcaneal insertion were higher in PwPHP [61]. Overall, the clinical comparison of PwPHP and controls showed the expected directions and magnitudes of differences supporting deployment of this protocol.

Considering that mechanical overload is thought to be a causal reason for PHP, and instrumented gait analysis the gold standard, we attempted to construct a graded loading challenge based on previous work to progressively challenge the load-bearing capacity of the plantar fascia by manipulating stride length and carried load [62]. If compressive or tensile load are aggravating factors for PHP, our results suggest the graded loaded challenge tasks may be a useful indicator of severity, particularly as the kinetic values show a graduated increase with task (Fig. 4).

#### Reliability

The ICC calculated for the overall risk factor scores such as pain duration and severity were excellent (ICC 0.92–0.94), which again suggests equivalence [15]. Previously validated questionnaire reliability was typically good to excellent (ICC 0.86–0.99), except one subscale of the FABQ (work) and FAOS (QoL). However, FAOS comparisons have previously shown remote use suitability [63]. This may indicate that our online questionnaire order, design and burden led to problems and requires further consideration. Finally, the biomechanical measures were repeated and demonstrated similar (Table 2) reliability to published work for kinetics [64]. Kinematic re-test reliability was not as comparable necessitating particular care with marker placement.

#### Limitations

The questionnaire design was kept as close to original as possible. However, some wording and layout had to be changed for the online mode, with these ‘faithful migrations’ [51] being acceptable but requiring the comprehensive testing detailed here. The Patient specific function scale (PSFS) had to be removed as the technology does not yet allow the responses from one questionnaire to be carried forward to follow-ups [65]. An open-ended question will be utilized instead of PSFS in the cohort study. We did not collect data on previous treatment in the feasibility study but have added this for the cohort study. This feasibility study did not implement or evaluate the follow-up process.

#### Feasibility lessons

In order to optimise questionnaire design, maximise data security, facilitate automated follow-up and enable eligibility screening we redesigned the survey to work on a different platform (SmartTrial 15,005-ST-0021, MEDEI ApS, Aalborg, Denmark) and pain mapping was moved to a high-resolution and detailed digital-body chart using the NavigatePain application Version 1 (Aalborg University, Aalborg, Denmark). In doing so, the repetition from the original survey was removed, without compromising questionnaire validity, and the process streamlined to reduce time and inconvenience. The streamlining included the addition of logic functions that enabled respondents to skip to a future question or page in the survey based on their answer to a previous close-ended question. Additionally, in the new versions participants will be able to resume and complete a survey having taken a break. Participants who are struggling with the initial questionnaires will also be offered support with completion if required. A decision to add health literacy assessment was taken in order to ensure population characteristics and data credibility. The clinical, ultrasound and biomechanical examinations were streamlined to reduce contact time, and improve ease of collection.

## Conclusion

Questionnaire administration by online methods is valid and reliable, therefore it could be ideal for remote monitoring of patients for clinical and research purposes, including our planned cohort study. A graded loading challenge designed to progressively increase kinetic load was shown to be a potentially useful assessment tool for plantar heel pain severity and worthy of further research. Hence, the questionnaire and graded loading challenge results in particular could be utilized by clinicians and researchers for a wide range of purposes. The cohort study is feasible.

## Supplementary Information

**Additional file 1.** Participants’ feedback from feasibility and pilot studies with some relevant quotes. (Key: Q = Quotation).

## Data Availability

The datasets analysed during the current study are available from the corresponding author on reasonable request.

## Abbreviations

BMI: Body mass index
FABQ: Fear-Avoidance Belief Questionnaire
FAOS: Foot and Ankle Outcome Score
GLC: Graded loading challenge
GPAQ: Global Physical Activity Questionnaire
ICC: Intraclass Correlation Coefficients
MLA: Medial longitudinal arch
MTPJ1: First metatarsophalangeal joint
OP: Other foot pain
PwOP: People with other foot pain
PHP: Plantar heel pain
PwPHP: People with plantar heel pain
PROMs: Patient reported outcome measures
PCS: Pain Catastrophizing Scale
vGRF: Vertical ground reaction force
