# Supplementary material for: Online questionnaire, clinical and biomechanical measurements for outcome prediction of plantar heel pain: feasibility for a cohort study
Source: J Foot Ankle Res. 2021 Apr 26;14:34. doi: 10.1186/s13047-021-00472-w (PMC8077700; doi:10.1186/s13047-021-00472-w)
Supplement: Supplementary file 1 — Additional file 1. Participants’ feedback from feasibility and pilot studies with some relevant quotes. (Key: Q = Quotation). [file 13047_2021_472_MOESM1_ESM.docx]

# Supplement

Supplement: 1 Participants’ feedbacks from feasibility and pilot studies with some relevant quoted answers. (Key: Q= Quotation)

| **Participants’ feasibility feedback** | | |
| --- | --- | --- |
| **Sub-themes** | **Findings** | **Illustrative Quotes** |
| First Thoughts on online survey | Comprehensive; seeks information on different facets; detailed; good layout, Long repetitive, time consuming, irrelevant questions, workable. Easy to use | Q1: The survey is comprehensive and seek information on different facets. However, it seems a bit long and I got lost halfway.  Q 2: The questionnaire is really long. I think the risk is to have a lot of withdraw… It should be shortened.  Q3: Why is it useful to know the highest studies degree, if the subject is married or not…  Q4: Easy to use just a little difficult as a control and definitely length which may put some people off. |
| Comments on pain chart reviewed | Workable, easy to use, imaginable, good to have different pain options. Difficult to draw on foot part,  Could be more detailed. | Q1: very good, it was not obvious at the start that the different pain options were available in the drop down, also the 0 to 10 pain scale did not open correctly on my page. The side bar was too narrow for the verbal description.  Q2: hey look fully workable to me! The only thing I found clunky was the body chart  Q3: What if patient has several pain types.  Q5: It was straight forward. Not clear how the pain scale would apply to more than one site of pain. Would be good to have enlarged diagrams of the smaller anatomical areas such as the foot. |
| Repetitions or difficult to understand | questions about emotional status, understandable, not too much repetition, | Q1: There are a lot of repetitive type questions on emotion etc. I understand these are part of validated questionnaires, but the last section is quite lengthy  Q2: No, nothing! |
| Any Error or anything not working properly | Skipping questions, going back previous page, irrelevant questions for control groups, dormant survey link, email title, grammar mistakes, VAS scale, | Q1: I completed this as a control with no current pain. Certainly, the earlier part of the questionnaire made this a little difficult / confusing. There may need to be some options for controls.  Q2: This looks like SPAM and not particularly good either. The email title does not refer to anything recognisable sorted out. The English is not great spelling and grammar checked  Q3: just the 0 to 10 slider description did not open correctly |
| Expectations / needed improvements | Be shorten, being informed people for next stage, more instructions and explanation for some medical terms, some question types are difficult to answers/ confusing options. Being easy to answers of questions in all devices, reminding of previous survey answers, pain drawing at the end of follow-up. | Q1: If it could be shorter, that would be better  Q2: The screen at the end of the eligibility could have warned me to expect another email  Q3: What do you mean by 'symptoms'  Q4: The date of birth system is quite clunky and could annoy someone.  Q5: in some questions you as patients if they had electrotherapy? Will a patient know what this is?  Q5: in the foot health status questionnaire the comment 'fairly many times' is a confusing term. Is this standard in this questionnaire?  Q6: Seeing the full question on screen is difficult  Q7: Adding several instructions or making buttons visually better might help.  Q8: In 3 months survey, I did not remember what I wrote before. If these could be listed, that might help.  Q9: Drawing at the end would be useful to compare my condition |
| Others | Comprehensive | Q1: well done, it is very comprehensive! |
